# Supplementary material for: The neuropeptide substance P regulates aldosterone secretion in human adrenals
Source: Nat Commun. 2020 May 29;11:2673. doi: 10.1038/s41467-020-16470-8 (PMC7260184; doi:10.1038/s41467-020-16470-8)
Supplement: Supplementary file 1 — Supplementary Information [file 41467_2020_16470_MOESM1_ESM.pdf]

The neuropeptide substance P regulates aldosterone secretion in human adrenals

Wils J, Duparc C et al.

Supplementary Methods: Clinical trial: Pharmacological testing design
Supplementary Discussion
Supplementary Table 1 Tachykinin effects on steroid secretion by adrenocortical cells
Supplementary Table 2 Clinical characteristics of healthy volunteers
Supplementary Table 3 Sequences and GenBank accession numbers of primers used for RT-PCR
Supplementary Table 4 Antibodies used for immunohistochemistry or immunohisto-fluorescence studies
Supplementary Table 5 Antibodies used for western blot analyses
Supplementary Fig. 1 Substance P in human adrenal arterioles
Supplementary Fig 2. Representative western blots showing Neurokinin type 1 receptor (NK1R) in adrenals samples.
Supplementary Fig. 3 NK1 receptor (NK1R) in human adrenal ganglia, arterioles and cortex
Supplementary Fig. 4 Dose-dependent effect of SP on ERK phosphorylation in cultured adrenocortical cells.
Supplementary Fig. 5 Effect of angiotensin II on the expression levels of genes encoding cholesterol transporter and steroidogenic enzymes
Supplementary Fig. 6 Simplified flowchart of the clinical trial testing the effect of oral administration of the NK1R antagonist aprepitant on corticosteroid production in healthy volunteers
Supplementary Fig. 7 Absence of effect of aprepitant on cortisol/ACTH ratio
Supplementary Fig. 8 Lack of effect of aprepitant on plasma ACTH and cortisol responses to insulin tolerance tests
Supplementary Fig. 9 Absence of effect of aprepitant on kaliemia
Supplementary Fig. 10 Absence of effect of aprepitant on blood pressure
Supplementary Fig. 11 Effect of aprepitant on plasma aldosterone and renin responses to metoclopramide
Supplementary note 1 Study protocol
Supplementary References

Supplementary Methods

Clinical trial: Pharmacological testing design

A metoclopramide stimulation test (10 mg iv; Primpéran, Synthélabo Laboratories, Meudon-la-Forêt, France) (test 2) and an insulin tolerance test (0,1 U kg^{-1} iv; Actrapid, Novo Nordisk, Courbevoie, France) (test 3) were performed in recumbency at D5 and D7, respectively (Supplementary Fig. 6). The impact of the procedures on the adrenocortical function was evaluated by measuring plasma aldosterone, cortisol, renin, electrolyte and/or ACTH levels before (t0) and every 30 min for 2 hrs after administration of metoclopramide, and before (t0) and every 15 min for 90 min after injection of insulin.

Supplementary Discussion

The NK1R antagonist aprepitant did not modify basal plasma cortisol and ACTH concentrations, as well as the cortisol to ACTH ratio, indicating that the adrenal sensitivity to ACTH is not influenced by the compound. The plasma cortisol and ACTH responses to the insulin stimulation test which mimics the action of stress on the hypothalamo-pituitary adrenal axis, remained unchanged under aprepitant treatment.

We have previously demonstrated that serotonin (5-HT), released by intraadrenal mast cells, stimulates aldosterone secretion through activation of the 5-HT4 receptors (5-HT4R) expressed by zona glomerulosa cells[1-3]. In this respect, administration of agonists of 5-HT4R, such as zacopride, cisapride and metoclopramide, has been reported to increase plasma aldosterone concentration in healthy volunteers[4-6]. In the gastrointestinal tract, it has been shown that SP is able to mediate the prokinetic action of 5-HT in response to activation of 5-HT4Rs[7]. These observations suggested that the action of 5-HT on mineralocorticoid function may be partly indirect *via* local release of SP. In order to explore this hypothesis, a pharmacological test was conducted with metoclopramide (10 mg iv), a partial agonist of 5-HT4R, in combination with aprepitant. Surprisingly, metoclopramide-induced aldosterone secretion, expressed as relative variation to basal level, was significantly enhanced by aprepitant. These data allow excluding an indirect action of the 5-HT4R agonist through SP nerve fibres. Conversely, the potentiation of the aldosterone response to metoclopramide by the NK1R antagonist appeared to be the consequence of an increase in renin production which was not observed under stimulation by metoclopramide alone, as previously reported[6]. It is conceivable that the elevation of plasma renin concentrations may result from an impact of the metoclopramide/aprepitant combination on the intrarenal blood flow.

Supplementary Table 1: Tachykinin effects on steroid secretion by adrenocortical cells

	Maximum efficacy [Emax]	Potency [EC50]
Effect of SP on cortisol secretion	184 ± 17 %	76.7 ± 0.3 nM
Effect of SP on aldosterone secretion	166 ± 8%	1.3 ± 0.3 nM
Effect of NKA on aldosterone secretion	177 ± 13%	27.3 ± 0.3 nM

Supplementary Table 2: Clinical characteristics of healthy volunteers

Healthy volunteers	20
Male	20
Age (year)	21.3±1.7
Body mass index (kg m^{-2})	22.4±1.7
Systolic blood pressure (mmHg)	127.9±11.3
Diastolic blood pressure (mmHg)	69.3±7.4
Cardiac frequency (bpm)	70±11

Supplementary Table 3: Sequences and GenBank accession numbers of primers used for RT-PCR

Gene	Primer	Sequence primer (5'-3')	Accession number	Product size (bp)
PPIA	F	ATGGCACTGGTGGCAAGTCC	NM_001300981.1	241
	R	TTGCCATTCCTGGACCCAAA	NM_021130.4	
TAC1	F	GACTGTCCGTCGCAAAATCC	NM_013996.2	150
	R	CCTCCTTGATCTGGTCGCTG	NM_013997.2<br>NM_013998.2<br>NM_003182.2	
TAC3	F	CTAGATCCCCTCCACTCGGT	NM_001178054.1<br>NM_013251.3	227
	R	TCCTCCTGTGGCTCCTTACA		
TAC4	F	CACTGAAGCAGAGACCTGGG	NM_170685.2	101
	R	TCCCCATCAGCCCAAAGAAC	NM_001077503.1<br>NM_001077504.1<br>NM_001077505.1<br>NM_001077506.1	
TACR1s		Origen Ref : HK210362	NM_015727.2	NP
TACR1l		Qiagen Ref : QT01009148	NM_001058.3	71
TACR2	F	GCTTCTACTCCACCGTCACCAT	NM_001057.2	148
	R	GCTACAAACATCACCGCGAGC		
TACR3	F	TCATAGCGAGTGGTACTTTGGC	NM_001059.2	147
	R	GACAGTCTGGGTTTCAAGGGA		
STAR	F	GGCTACTCAGCATCGACCTC	NM_000349.2	250
	R	CATCCCACTGTCACCAGATG		
HSD3B2	F	TTGGACAAGGCCTTCAGACA	NM_000198.3	150
	R	ACAGGCGGTGTGGATGAC	NM_001166120.1	
CYP21A2	F	GAGTTCTGTGAGCGCA	NM_000500.7<br>NM_001128590.3	201
	R	CACGTCCACAATTTGGAT		
CYP11B2	F	TCCAGGTGTGTTCAGTAGTTCC	NM_000498.3	146
	R	GAAGCCATCTCTGAGGTCTGTG		

NP, not provided by the manufacturer.

Supplementary Table 4: Antibodies used for immunohistochemistry or immunohistofluorescence studies

Antibody	Source	Clonality	Host	Dilution	Antigen retrieval	Validation on tissues	Ref
Aldosterone Synthase	Dr Gomez-Sanchez	Monoclonal clone 41	mouse	1/100	Tris EDTA pH 9	Human adrenal	8
Substance P	MM-0001-1 Interchim	Monoclonal clone NC1/34	rat	1/25	Citrate pH 6	Human Achilles tendon	9
NK1 receptor against the 2nd extracellular loop	T5950 Sigma-Aldrich	Polyclonal Affinity isolated antibody	rabbit	1/200	Citrate pH 6	Human ovary	10
NK1 receptor against the 211-260 amino acid region	SAB4502913 Sigma-Aldrich	Polyclonal Affinity isolated antibody	rabbit	1/4000	Citrate pH 6	Human blood vessel	11 12 13
NK1 receptor against the 387-407 amino acid region	PA3-301 ThermoFisher	Polyclonal	rabbit	1/800	Citrate pH 6	Human spinal cord	14
Protein gene product 9.5 (PGP9.5)	AB1761 Merck	Polyclonal Affinity purified antibody	rabbit	1/250	Citrate pH 6	Human spinal cord	15
Tyrosine hydroxylase	MAB 318, Merck Millipore	Monoclonal clone LNC1	mouse	1/100	Citrate pH 6	Human substantia nigra	16
Choline acetyl transferase	AB144P Merck Millipore	Polyclonal Affinity purified antibody	goat	1/200	Citrate pH 6	Human cardiac neurons	17
Anti-rat IgG/HRP	P045001 Dako		rabbit	1/100			
EnVision rabbit IgG/HRP	K4002 Dako		goat	Ready to use			
Alexa fluor 488 anti-rat IgG	A-21208 Life Technologies		donkey	1/300			
Alexa fluor 594 anti-rabbit IgG	A-21207 Life Technologies		donkey	1/300			
Alexa fluor 594 anti-mouse IgG	A-21203 Life Technologies		donkey	1/300			
Alexa fluor 594 anti-goat IgG	A-11058 Life Technologies		donkey	1/300			
Alexa fluor 647 anti-mouse IgG	FP-SC4110 Interchim		donkey	1/300			

Supplementary Table 5: Antibodies used for western blot analyses

Antibodies	Source	Clonality	Host	Dilution	Validation on tissues
NK1 receptor against the 2nd extracellular loop	T5950 Sigma-Aldrich	Polyclonal Affinity isolated antibody	rabbit	1/1000	Human ovary[10]
NK1 receptor against the 211-260 amino acid region	SAB4502913 Sigma-Aldrich	Polyclonal Affinity isolated antibody	rabbit	1/500	Human blood vessel[11]
NK1 receptor against the 387-407 amino acid region	PA3-301 ThermoFisher	Polyclonal	rabbit	1/2000	Human spinal cord[14]
p44/42 MAPK (ERK1/2)	4696 Cell Signaling	Monoclonal	mouse	1/1000	Different cell lines*
phospho-p44/42 MAPK (ERK1/2)	4370 Cell Signaling	Monoclonal	rabbit	1/1000	Different cell lines**
Vinculine	MCA465GA Biorad	Monoclonal	mouse	1/1000	Hela cells***
anti-rabbit IgG, (H1L), peroxydase conjugated	31460 Pierce		goat	1/10000	
anti-mouse IgG, (H1L), peroxydase conjugated	31430 Pierce		goat	1/10000	

*https://www.cellsignal.com/products/primary-antibodies/p44-42-mapk-erk1-2-l34f12-mouse-mab/4696
**https://www.cellsignal.com/products/primary-antibodies/phospho-p44-42-mapk-erk1-2-thr202-tyr204-d13-14-4e-xp-rabbit-mab/4370
*** https://www.bio-rad-antibodies.com/monoclonal/human-vinculin-antibody-v284-mca465.html?f=purified

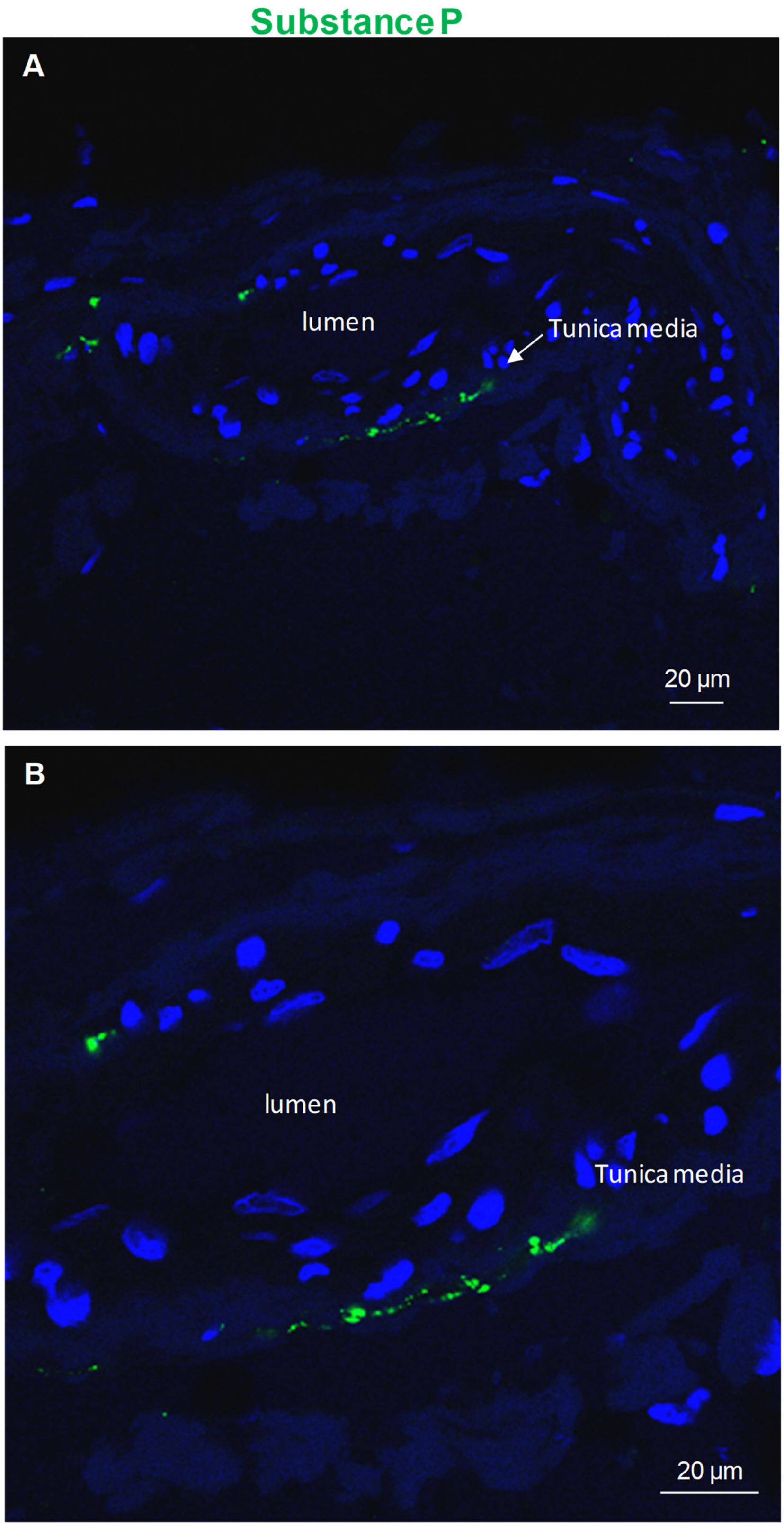

Supplementary Figure 1. Substance P in human adrenal arterioles. **A, B** Immunofluorescence detection of SP (green) in the wall of arterioles irrigating the adrenal cortex at low (A) and high (B) magnifications (Microphotographs representative of n = 20 independent adrenals).

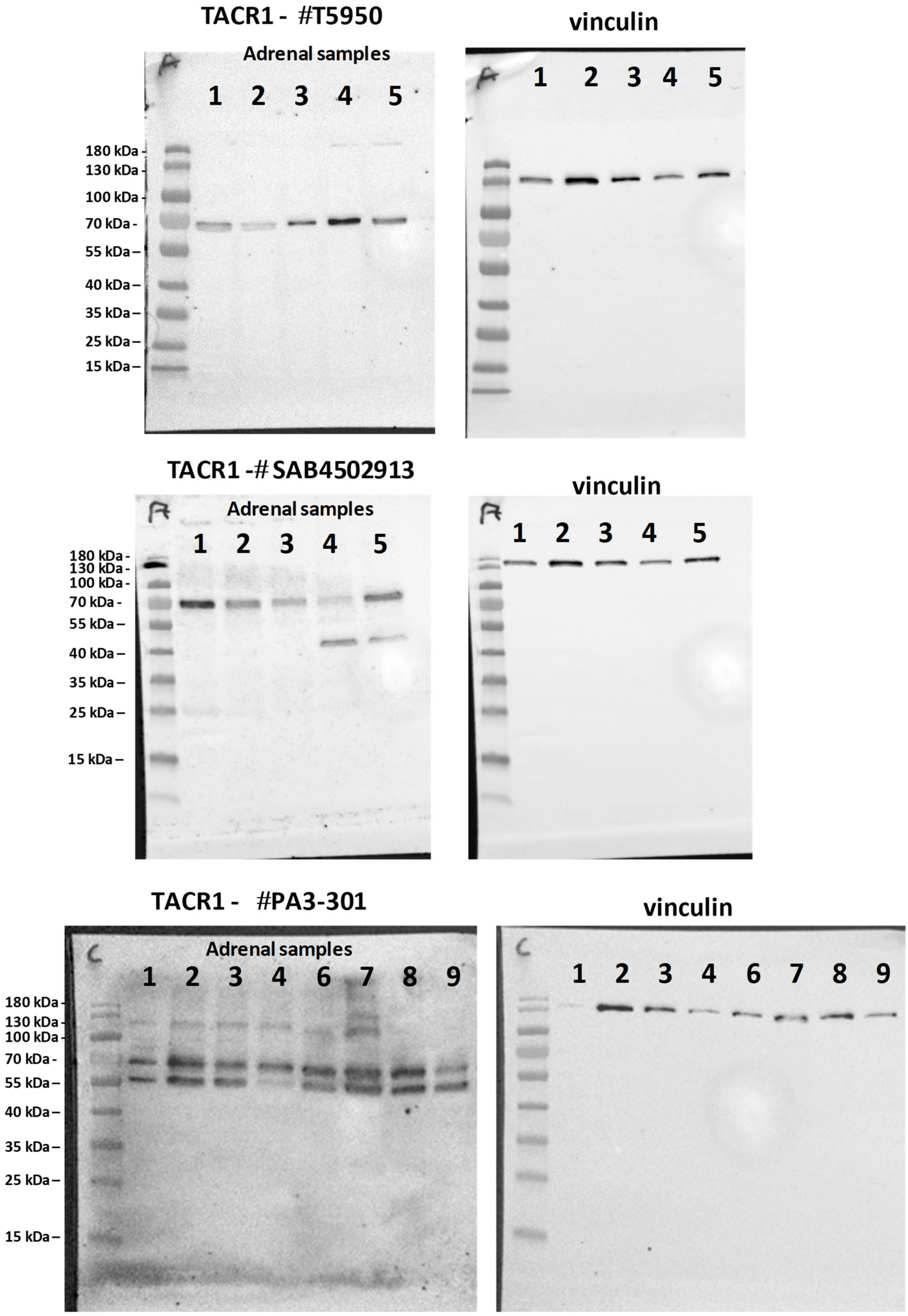

Supplementary Figure 2. Neurokinin type 1 receptor (NK1R) in adrenals. Representative western blots showing NK1R (left panels) revealed by three different antibodies (T5950, SAB4502913, PA3-301) in n= 5 (upper and middle panels) and 8 (lower panels) independent adrenal samples. Vinculin used as a loading control was revealed on the same membranes after remove stripping procedure (right panels).

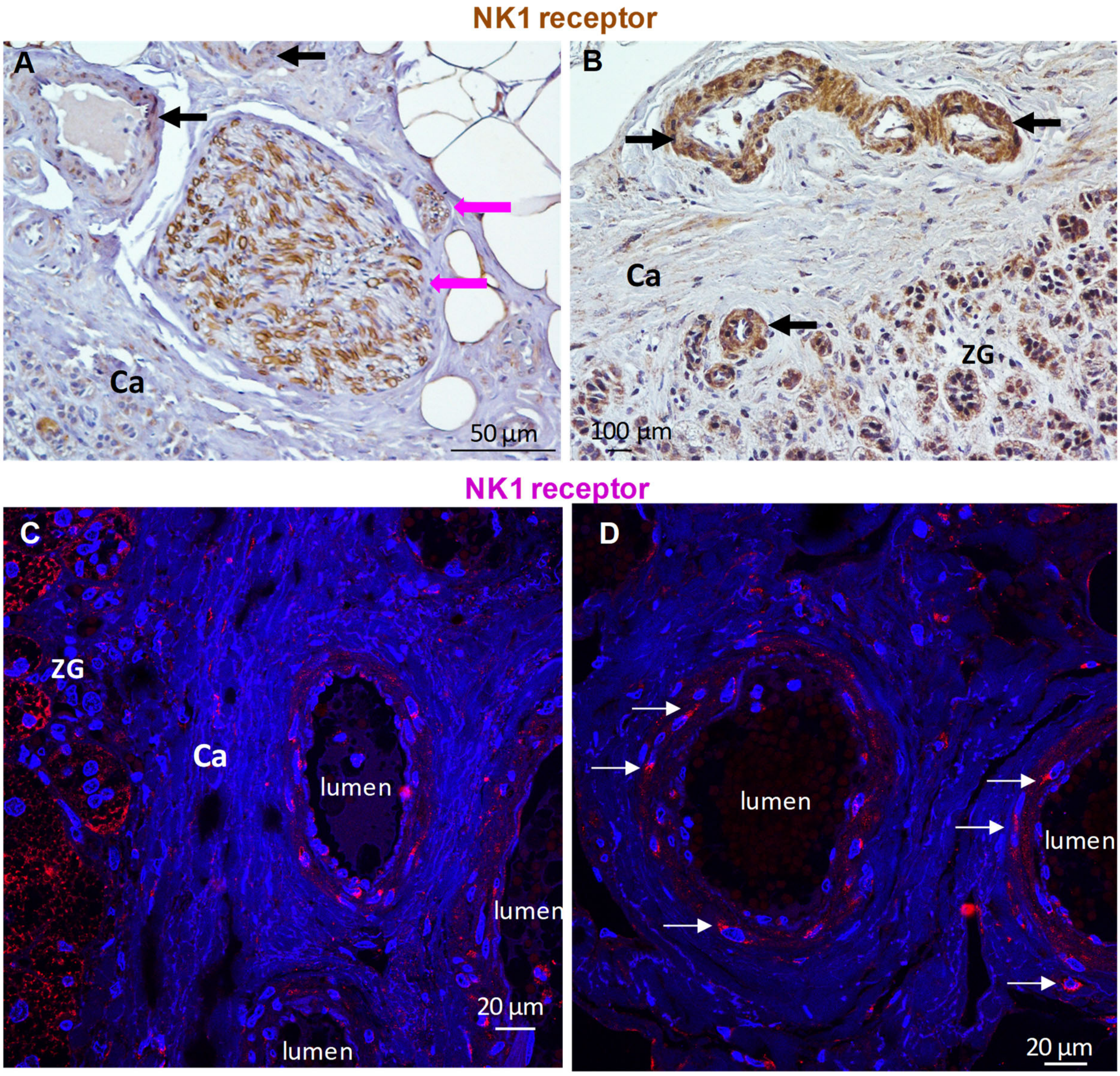

Supplementary Figure 3. NK1 receptor (NK1R) in human adrenal ganglia, arterioles and cortex. **A, B,** Immunohistochemical detection of NK1R immunoreactivity in ganglia at the periphery of adrenal (pink arrows), the wall of arterioles irrigating the adrenal gland (black arrows), and zona glomerulosa (ZG) in the subcapsular region of the cortex (microphotographs representative of n = 19 independent adrenals). **C, D** Immunofluorescence detection of NK1R immunoreactivity in the wall of arterioles irrigating the adrenal gland, and zona glomerulosa (ZG) at low (C) and high (D) magnifications (microphotographs representative of n = 4 independent adrenals). White arrows indicate NK1R-positive muscle cells in tunica media (C, D). Ca, capsule.

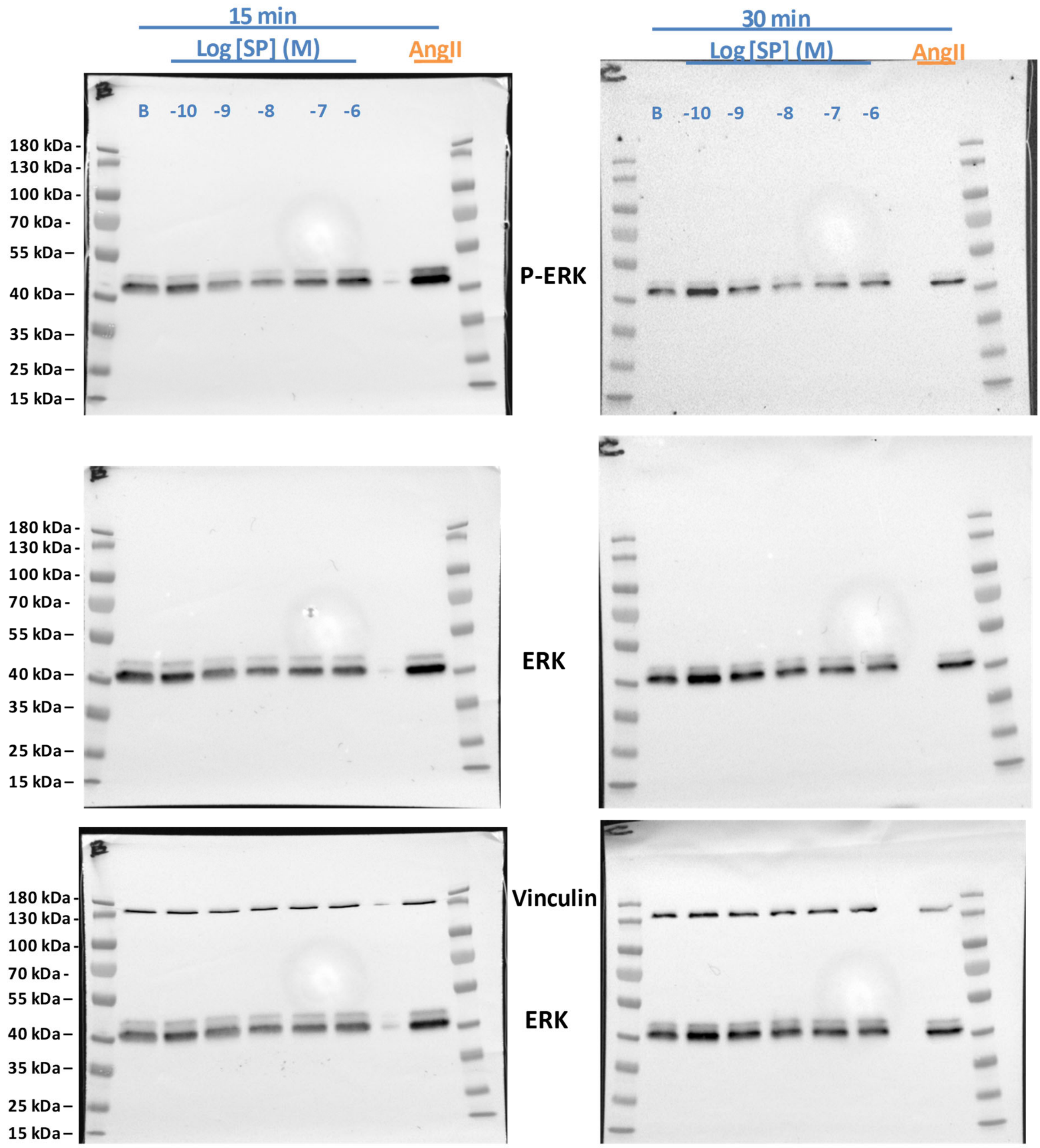

Supplementary Figure 4. Dose-dependent effect of SP on ERK phosphorylation in cultured adrenocortical cells. Representative western blots showing the effect of increasing doses of SP (from 10^{-10} to 10^{-6} M) administrated for 15 (left panels) or 30 min (right panels) on phospho-ERK (upper panels) and ERK (middle panels) levels (n = 3 independent experiments). Phospho-ERK and ERK immunolabelings were successively performed on the same membranes after remove stripping procedure. The blots showing phospho-ERK or ERK at 15 and 30 min were processed in parallel with those of Fig. 5C (5 min). They show samples derived from the same culture experiment. Ang II (10^{-6} M) and vinculin (lower panels) were used as positive and loading controls, respectively.

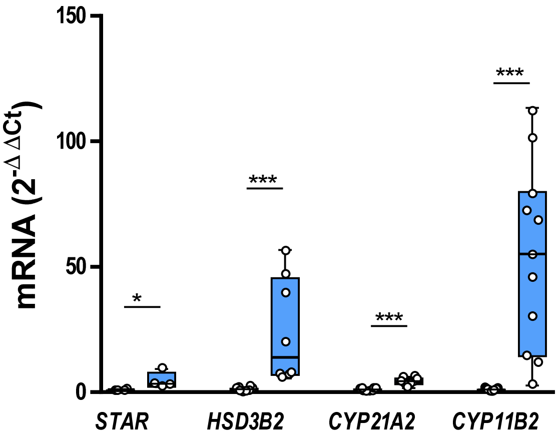

Supplementary Figure 5. Effect of angiotensin II on the expression levels of genes encoding cholesterol transporter and steroidogenic enzymes. Angiotensin II (AngII; 10^{-6} M) was applied on cultured human adrenocortical cells for 24 hrs. mRNA expression levels were normalized to PPIA. Data are expressed as median ± IQR, minima and maxima (n=4, 8, 8 and 11 cultures for *STAR*, *HSD3B2*, *CYP21A2* and *CYP11B2*, respectively). They were analyzed by two-tailed Mann Whitney test (p=0.03 for *STAR*, p=0.0002 for *HSD3B2*, p= 0.0002 for *CYP21A2* and p<0.0001 for *CYP11B2*). *, $p < 0.05$; ***, $p < 0.001$.

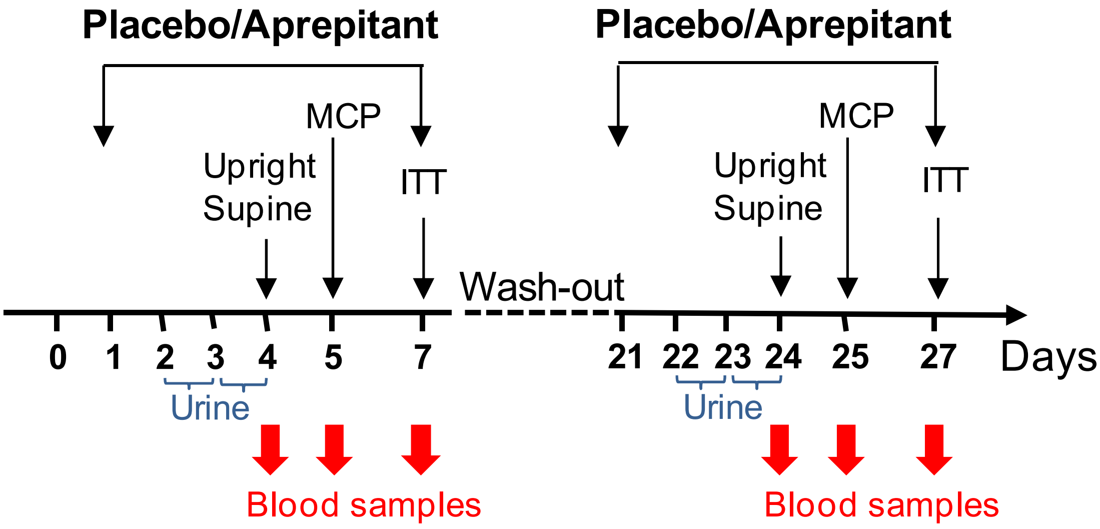

Supplementary Figure 6. Simplified flowchart of the clinical trial testing the effect of oral administration of the NK1R antagonist aprepitant on corticosteroid production in healthy volunteers. Healthy male volunteers underwent two one-week treatment (placebo and/or aprepitant in a random order) periods separated by a two-week wash-out period. Aprepitant was administered at the doses of 125 mg at day 1 and 80 mg per day for the six other days. Subjects underwent a series of tests including upright and supine positions at day 4 (D4) and D24, metoclopramide (MCP; 10 mg iv) stimulation tests at D5 and D25, and insulin tolerance tests (ITT; 0,1 U kg^{-1} iv) at D7 and D27. Blue braces indicate 24-h urine collections. Red arrows indicate blood samples for analysis.

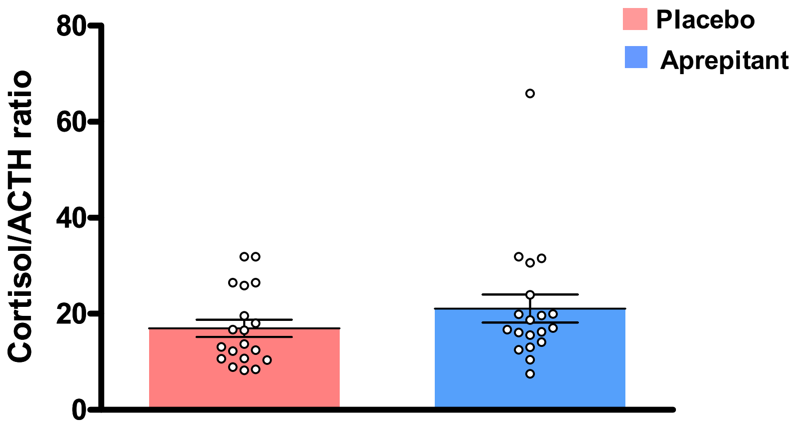

Supplementary Figure 7. Absence of effect of aprepitant on cortisol/ACTH ratio. Plasma cortisol and ACTH levels were measured in healthy volunteers under placebo or aprepitant in supine position at D7 (day 7 or 27; CI95%: 13.2-20.8 *versus* 14.9-27.3; p=0.27; n=20). Data are expressed as means ± SEM. They were analyzed by non-parametric Wilcoxon/mid rank matched pairs test.

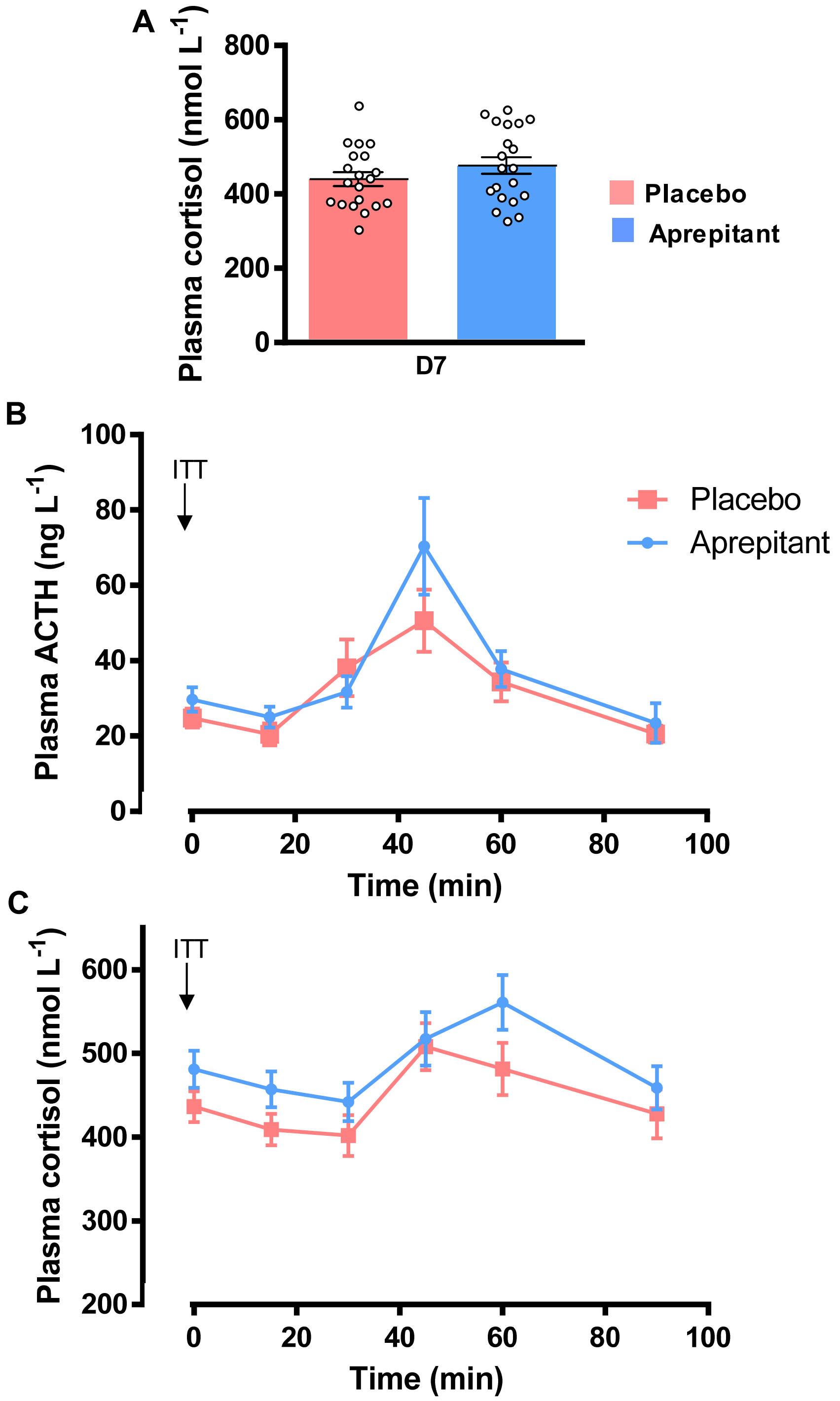

Supplementary Figure 8. Lack of effect of aprepitant on plasma ACTH and cortisol responses to insulin tolerance tests. **A,** Absence of effect of aprepitant on plasma cortisol levels at D7 (day 7 or 27; CI95%: 401-479 *versus* 429-524; *p*= 0.25; n=20). Data are expressed as means ± SEM. They were analyzed by non-parametric Wilcoxon/mid rank matched pairs test. **B, C** Kinetics of plasma ACTH (B) and cortisol (C) responses to insulin tolerance tests (ITT; 0,1 U/kg iv) in 20 healthy volunteers under placebo or aprepitant at D7 (day 7 or 27). Aprepitant did not modify the ACTH (F=0.69; DFn=1; DFd=190; *p*=0.41) and cortisol (F=2.51; DFn=1; DFd=190; p=0.12) responses to the insulin stimulation test. Data are represented as mean ± SEM and analyzed by Two-way ANOVA. F: distribution; DFn: degrees of freedom for groups; DFd: degrees of freedom for samples.

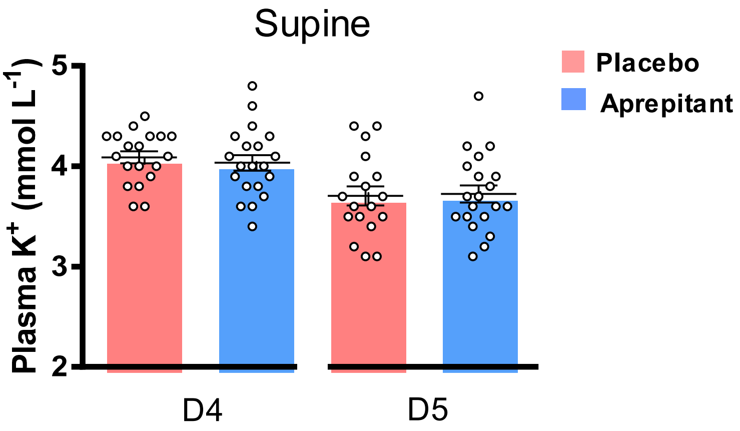

Supplementary Figure 9. Absence of effect of aprepitant on kaliemia. Potassium levels were measured in healthy volunteers under placebo or aprepitant in supine position at both D4 (day 4 or 24; CI95%: 3.9-4.2 *versus* 3.9-4.2; p= 0.20; n=20) and D5 (day 5 or 25; CI95%: 3.5-3.9 *versus* 3.5-3.9; p=0.99; n=20). Data are expressed as means ± SEM. Data were analyzed by non-parametric Wilcoxon/mid rank matched pairs test.

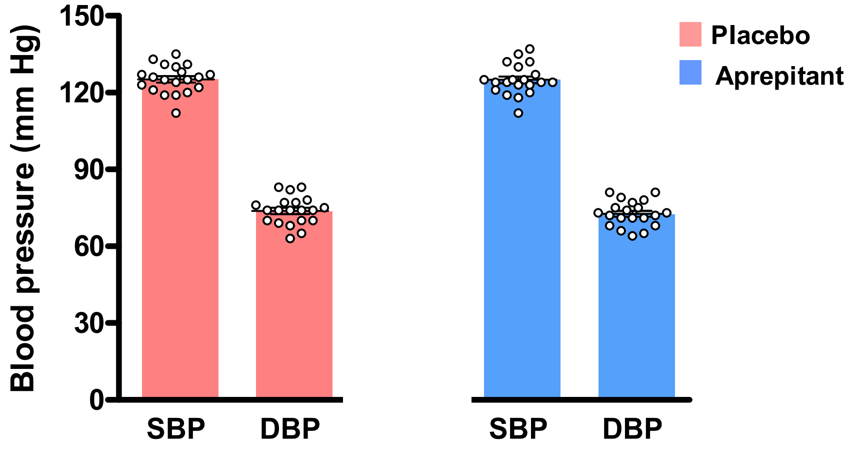

Supplementary Figure 10. Absence of effect of aprepitant on blood pressure. Systolic (SBP) and diastolic (DBP) blood pressures were measured in healthy volunteers under placebo or aprepitant at D3/D4 (days 3/4 or 23/24) by using 24-h ambulatory blood pressure monitoring. SBP: CI95%: 122-127 *versus* 122-127; p=0.95; n=20. DBP: CI95%: 71-76 *versus* 70-75; p=0.39; n=20. Data are expressed as means ± SEM. They were analyzed by non-parametric Wilcoxon/mid rank matched pairs test.

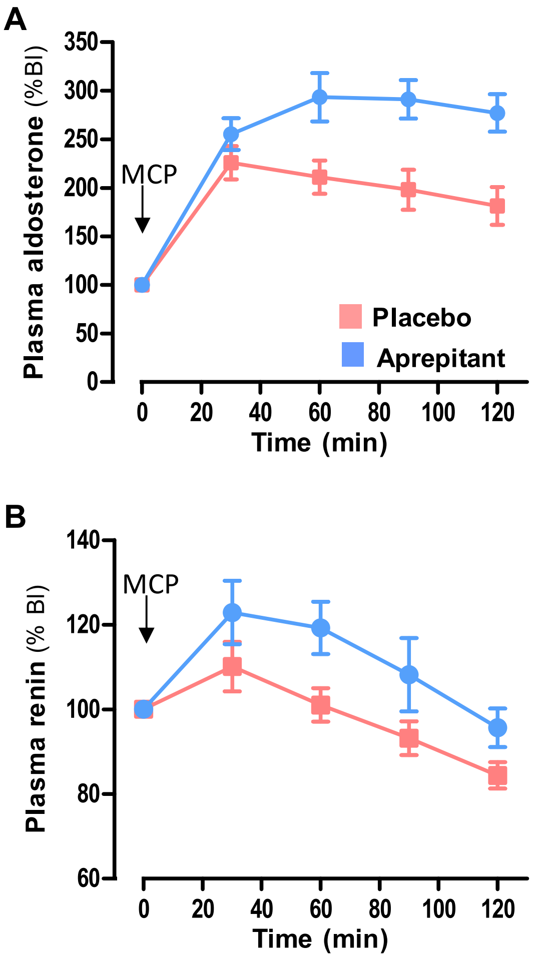

Supplementary Figure 11. Effect of aprepitant on plasma aldosterone and renin responses to metoclopramide. **A, B** Kinetics of plasma aldosterone (A) and renin (B) responses to metoclopramide (MCP, 10 mg iv) in 20 healthy volunteers under placebo or aprepitant at D5 (day 5 or 25). Aprepitant enhanced the effects of metoclopramide on aldosterone (F=10.49; DFn=1; DFd=152; *p*=0.002; n=20) and renin (F=6.21; DFn=1; DFd=152; *p*=0.017) plasma levels. Variations of plasma concentrations are expressed as % basal level (Bl). Data are expressed as means ± SEM and analyzed by Two-way ANOVA. F: distribution; DFn: degrees of freedom for groups; DFd: degrees of freedom for samples.

Supplementary note 1 Study protocol

EudraCT: 2008-003367-40

Annex 1: Clinical trial Application Form

**REQUEST FOR AUTHORISATION OF A CLINICAL TRIAL ON A MEDICINAL PRODUCT FOR HUMAN USE TO THE COMPETENT AUTHORITIES AND FOR OPINION OF THE ETHICS COMMITTEES IN THE COMMUNITY**

To be filled in by the applicant
The questions in this form for the request for authorisation from the Competent Authority are also relevant for the opinion from an Ethics Committee (it represents module 1 of the form for applying to an ethics committee) and can be used as part of that application. Please indicate the relevant purpose in a box below.

REQUEST FOR AUTHORISATION TO THE COMPETENT AUTHORITY: **Yes •**
REQUEST FOR OPINION OF THE ETHICS COMMITTEE: **No •**

A. TRIAL IDENTIFICATION

A.1	Member State in which the submission is being made:	**France - ANSM**
A.2	EudraCT number:	**2008-003367-40**
A.3	Full title of the trial:	
	**English**	**Pilot study of the effect of a substance P antagonist, aprepitant, on aldosterone and cortisol secretions in healthy volunteers**
	**French**	**ETUDE PILOTE DE L'EFFET D'UN ANTAGONISTE DE LA SUBSTANCE P, L'APREPITANT, SUR LES SECRETIONS D'ALDOSTERONE ET DE CORTISOL CHEZ LE VOLONTAIRE SAIN**
A.3.1	Title of the trial for lay people, in easily understood, i.e. non-technical, language:	
	**English**	**Pilot study of the effect of a substance P antagonist, aprepitant, on aldosterone and cortisol secretions in healthy volunteers**
	**French**	**ETUDE PILOTE DE L'EFFET D'UN ANTAGONISTE DE LA SUBSTANCE P, L'APREPITANT, SUR LES SECRETIONS D'ALDOSTERONE ET DE CORTISOL CHEZ LE VOLONTAIRE SAIN**
A.3.2	Name or abbreviated title of the trial where available:	
	**English**	**APHOS**
A.4	Sponsor's protocol code number, version and date[1]:	
A.4.1	Sponsor's protocol code number:	**2007/049/HP**
A.4.2	Sponsor's protocol version:	**1**
A.4.3	Sponsor's protocol date:	**2008-12-01**
A.5	Additional international study identifiers (e.g. WHO, ISRCTN[2] , US NCT Number[3]) if available	
A.5.1	ISRCTN number:	
A.5.2	US NCT number:	
A.5.3	WHO Universal Trial Number (UTN):	
A.5.4	Other Identifier:	
A.6	Is this a resubmission?	**No •**
	If 'Yes', indicate the resubmission letter[4]:	**First Submission**
A.7	Is the trial part of an agreed Paediatric Investigation Plan?	**No •**
A.8	EMA Decision number of Paediatric Investigation Plan:	

B. IDENTIFICATION OF THE SPONSOR RESPONSIBLE FOR THE REQUEST

**B.1**	**SPONSOR**	
B.1.1	Name of organisation:	**CHU de Rouen**
B.1.2	Name of the person to contact:	
B.1.2.1	Given name	**Francois**
B.1.2.2	Middle name	
B.1.2.3	Family name	**TEILLARD**
B.1.3	Address:	
B.1.3.1	Street address	**1 rue de Germont**
B.1.3.2	Town/city	**Rouen**
B.1.3.3	Post code	**76031**
B.1.3.4	Country	**France**
B.1.4	Telephone number:	**+33 232 88 8265**
B.1.5	Fax number:	**+33 232 88 8287**
B.1.6	E-mail:	**secretariat.drc@chu-rouen.fr**

**B.2**	**LEGAL REPRESENTATIVE[5] OF THE SPONSOR IN THE COMMUNITY FOR THE PURPOSE OF THIS TRIAL** (if different from the sponsor)
B.2.1	Name of organisation:
B.2.2	Name of person to contact:
B.2.2.1	Given name
B.2.2.2	Middle name
B.2.2.3	Family name
B.2.3	Address:
B.2.3.1	Street address
B.2.3.2	Town/city
B.2.3.3	Post code
B.2.3.4	Country
B.2.4	Telephone number:
B.2.5	Fax number:
B.2.6	E-mail:

**B.3**	**STATUS OF THE SPONSOR:**	
B.3.1	Commercial:	**No •**
B.3.2	Non commercial:	**Yes •**

**B.4**	**Source(s) of Monetary or Material Support for the clinical trial (repeat as necessary):**	
B.4.1	Name of organisation:	**CHU de Rouen**
B.4.2	Country:	**France**

**B.5**	**Contact point[6] designated by the sponsor for further information on the trial**	
B.5.1	Name of organisation:	**CHU de Rouen**
B.5.2	Functional name of contact point (e.g. "Clinical Trial Information Desk"):	**TEILLARD**
B.5.3	Address:	
B.5.3.1	Street address	**1 rue de Germont**
B.5.3.2	Town/city	**Rouen**
B.5.3.3	Post code	**76031**
B.5.3.4	Country	**France**
B.5.4	Telephone number:	**+33 232 88 8265**
B.5.5	Fax number:	**+33 323 88 8287**
B.5.6	E-mail: (use a functional e-mail address rather than a personal one)	**secretariat.drc@chu-rouen.fr**

C. APPLICANT IDENTIFICATION, (please tick the appropriate box)

**C.1**	**REQUEST FOR THE COMPETENT AUTHORITY**		
C.1.1	Sponsor		**Yes •**
C.1.2	Legal representative of the sponsor		
C.1.3	Person or organisation authorised by the sponsor to make the application		
C.1.4	Complete the details of the applicant below even if they are provided elsewhere on the form:		
C.1.4.1	Name of Organisation:	**CHU de Rouen**	
C.1.4.2	Name of contact person:		
C.1.4.2.1	Given name	**Francois**	
C.1.4.2.2	Middle name		
C.1.4.2.3	Family name	**TEILLARD**	
C.1.4.3	Address:		
C.1.4.3.1	Street address	**1 rue de Germont**	
C.1.4.3.2	Town/city	**Rouen**	
C.1.4.3.3	Post code	**76031**	
C.1.4.3.4	Country	**France**	
C.1.4.4	Telephone number:	**+33 232 88 8265**	
C.1.4.5	Fax number:	**+33 232 88 8287**	
C.1.4.6	E-mail:	**secretariat.drc@chu-rouen.fr**	
C.1.5	Request to receive a copy of CTA data as XML:		
C.1.5.1	Do you want a copy of the CTA form data saved on EudraCT as an XML file?	**No •**	
C.1.5.1.1	If Yes provide the e-mail address(es) to which it should be sent (up to 5 addresses):		
C.1.5.1.2	Do you want to receive this via password protected link(s)[7]?	**No •**	
If you answer No to question C.1.5.1.2 the .xml file will be transmitted by less secure e-mail link(s)			

D. INFORMATION ON EACH IMP

Information on each 'bulk product' before trial-specific operations (blinding, trial specific packaging and labelling) should be provided in this section for each investigational medicinal product (IMP) being tested including each comparator and each placebo, if applicable. ***For placebo go directly to D.8.*** *If the trial is performed with several products use extra pages and give each product a sequential number in D.1.1. If the product is a combination product, information should be given for each active substance.*

**D.1**	**IMP IDENTIFICATION**	
Indicate which of the following is described below, then repeat as necessary for each of the numbered IMPs to be used in the trial (assign numbers from 1-n):		
D.1.1	This refers to the IMP number:	**PR1**
D.1.2	IMP being tested	**Yes •**
D.1.3	IMP used as a comparator	**No •**

**D.2**	**STATUS OF THE IMP**	
D.2.1	Has the IMP to be used in the trial a marketing authorisation?	**Yes •**
**If the IMP has a marketing authorisation in the Member State concerned by this application, but the trade name and marketing authorisation holder are not fixed in the protocol, go to section D.2.2.**		
D.2.1.1	If 'Yes', specify the product to be used in the clinical trial:	
D.2.1.1.1	Trade name **EMEND**	
D.2.1.1.1.1	EV Product Code (where applicable)	
D.2.1.1.2	Name of the Marketing Authorisation Holder:	**Merck Sharp &Dohme Ltd**
D.2.1.1.3	Marketing Authorisation number (if Marketing Authorisation granted by a Member State):	**564 930.8 and 564 931.4**
D.2.1.1.4	Is the IMP modified in relation to its Marketing Authorisation?	**No •**
D.2.1.1.4.1	If 'Yes', please specify:	
D.2.1.2	The country that granted the Marketing Authorisation	**France**
D.2.1.2.1	Is this the Member State concerned with this application?	**Yes •**

D.2.2	Situations where an IMP to be used in the CT has a Marketing Authorisation in the Member State concerned, but the protocol allows that any brand of the IMP with a Marketing Authorisation in that Member State be administered to the trial subjects and it is not possible to clearly identify the IMP(s) in advance of the trial start	
D.2.2.1	In the protocol, is treatment defined only by active substance?	**Yes •**
D.2.2.1.1	If 'Yes', give active substance in D.3.8 or D.3.9	
D.2.2.2	In the protocol, do treatment regimens allow different combinations of marketed products used according to local clinical practice at some or all investigator sites in the MS?	**No •**
D.2.2.2.1	If 'Yes', give active substance in D.3.8 or D.3.9	
D.2.2.3	The products to be administered as IMPs are defined as belonging to an ATC group[9]	**No •**
D.2.2.3.1	If 'Yes', give the ATC group of the applicable authorised codes in the ATC code field (level 3 or the level that can be defined) in D.3.3	
D.2.2.4	Other:	**No •**
D.2.2.4.1	If 'Yes', please specify:	

D.2.3	IMPD submitted:	
D.2.3.1	Full IMPD:	**No •**
D.2.3.2	Simplified IMPD:	**No •**
D.2.3.3	Summary of product characteristics (SmPC) only:	**Yes •**
D.2.4	Has the use of the IMP been previously authorised in a	**No •**

D.2.4.1	clinical trial conducted by the sponsor in the Community?<br>If 'Yes' specify which Member States:	
D.2.5	Has the IMP been designated in this indication as an orphan drug in the Community?	**No •**
D.2.5.1	If 'Yes', give the orphan drug designation number[10]**:**	

D.2.6	Has the IMP been the subject of scientific advice related to this clinical trial?	**No •**
D.2.6.1	If 'Yes' to D.2.6, please indicate source of advice and provide a copy in the CTA request:	
D.2.6.1.1	CHMP[11]?	**No •**
D.2.6.1.2	National Competent Authority?	**No •**

**D.3**	**DESCRIPTION OF THE IMP**	
D.3.1	Product name where applicable[12]:	
D.3.2	Product code where applicable[13]:	
D.3.3	ATC codes, if officially registered[14]:	**A04AD12**
D.3.4	Pharmaceutical form (use standard terms):	**Capsule**
D.3.4.1	Is this a specific paediatric formulation?	**No •**
D.3.5	Maximum duration of treatment of a subject according to the protocol:<br>**1 capsule per day during 1 week**	
D.3.6	Dose allowed:	
D.3.6.1	For first trial only:	
	Specify per day or total	**Not Answered •**
	Specify total dose (number and unit):	
	Route of administration (relevant to the first dose):	
D.3.6.2	For all trials	
	Specify per day or total	**Total •**
	Specify total dose (number and unit):	**1 week treatment duration : 125 mg/day at day 1 and 80 mg/day during 6 days mg milligram(s)**
	Route of administration (relevant to the maximum dose):	**Oral use**
D.3.7	Routes of administration (use standard terms):	**Oral use**

D.3.8	Name of each active substance (INN or proposed INN if available):<br>**APREPITANT**	
D.3.9	Other available name for each active substance (provide all available):	
D.3.9.1	CAS[15] number	**170729-80-3**
D.3.9.2	Current sponsor code	
D.3.9.3	Other descriptive name	
D.3.9.4	EV Substance code	**SUB20017**
D.3.9.5	Full Molecular formula	
D.3.9.6	Chemical/biological description of the Active Substance	
D.3.10	Strength (specify all strengths to be used):	
D.3.10.1	Concentration unit:	**mg milligram(s)**
D.3.10.2	Concentration type ("exact number", "range", "more than" or "up to"):	**up to**
D.3.10.3	Concentration (number).	**125**

D.3.11	Type of IMP	
Does the IMP contain an active substance:		
D.3.11.1	Of chemical origin?	**Yes •**
D.3.11.2	Of biological / biotechnological origin (other than Advanced Therapy IMP (ATIMP)?	**No •**

Is this a:

D.3.11.3	Advanced Therapy IMP (ATIMP)?	**No •**
D.3.11.3.1	Somatic cell therapy medicinal product[16]?	**No •**
D.3.11.3.2	Gene therapy medicinal product[17]?	**No •**
D.3.11.3.3	Tissue Engineered Product[18]?	**No •**
D.3.11.3.4	Combination ATIMP (i.e. one involving a medical device[19])?	**No •**
D.3.11.3.5	Has the Committee on Advanced Therapies issued a classification for this product?	**No •**
D.3.11.3.5.1	If 'Yes' please provide that classification and its reference number:	
D.3.11.4	Combination product that includes a device, but does not involve an Advanced Therapy?	**No •**
D.3.11.5	Radiopharmaceutical medicinal product?	**No •**
D.3.11.6	Immunological medicinal product (such as vaccine, allergen, immune serum)?	**No •**
D.3.11.7	Plasma derived medicinal product?	**No •**
D.3.11.8	Extractive medicinal product?	**No •**
D.3.11.9	Recombinant medicinal product?	**No •**
D.3.11.10	Medicinal product containing genetically modified organisms?	**No •**
D.3.11.10.1	Has the authorisation for contained use or release been granted?	**No •**
D.3.11.10.2	Is it pending?	**No •**
D.3.11.11	Herbal medicinal product?	**No •**
D.3.11.12	Homeopathic medicinal product?	**No •**
D.3.11.13	Another type of medicinal product?	**No •**
D.3.11.13.1	If 'another type of medicinal product' specify the type of medicinal product:	
D.3.12	Mode of action (*free text*[20])	
D.3.13	Is it an IMP to be used in a first-in-human clinical trial?	**No •**
D.3.13.1	If 'Yes', are there risk factors identified, according to the guidance FIH?[21]	

**D.4**	**SOMATIC CELL THERAPY INVESTIGATIONAL MEDICINAL PRODUCT (NO GENETIC MODIFICATION)**	
D.4.1	Origin of cells	
D.4.1.1	Autologous	**No •**
D.4.1.2	Allogeneic	**No •**
D.4.1.3	Xenogeneic	**No •**
D.4.1.3.1	If 'Yes', specify the species of origin:	
D.4.2	Type of cells	
D.4.2.1	Stem cells	**No •**
D.4.2.2	Differentiated cells	**No •**
D.4.2.2.1	If 'Yes', specify the type (e.g. keratinocytes, fibroblasts, chondrocytes...):	
D.4.2.3	Others:	**No •**
D.4.2.3.1	If others, specify:	

**D.5**	**GENE THERAPY INVESTIGATIONAL MEDICINAL PRODUCTS**	
D.5.1	Gene(s) of interest:	
D.5.2	In vivo gene therapy:	**No •**
D.5.3	Ex vivo gene therapy:	**No •**
D.5.4	Type of gene transfer product	
D.5.4.1	Nucleic acid (e.g. plasmid): If 'Yes', specify if:	**No •**
D.5.4.1.1	Naked:	**No •**

D.5.4.1.2	Complexed	**No •**
D.5.4.2	Viral vector:	**No •**
D.5.4.2.1	If 'Yes', specify the type: adenovirus, retrovirus, AAV, ...:	
D.5.4.3	Others	**No •**
D.5.4.3.1	If others, specify:	
D.5.5	Genetically modified somatic cells:	**No •**
If 'Yes', specify the origin of the cells:		
D.5.5.1	Autologous:	**No •**
D.5.5.2	Allogeneic:	**No •**
D.5.5.3	Xenogeneic:	**No •**
D.5.5.3.1	If 'Yes', specify the species of origin:	
D.5.5.4	Specify type of cells (hematopoietic stem cells...):	

**D.6**	**TISSUE ENGINEERED PRODUCT**	
The indication which determines that this is a Tissue Engineered Product as opposed to a Cell Therapy product is given in section E.1.1.		
D.6.1	Origin of cells	
D.6.1.1	Autologous	**No •**
D.6.1.2	Allogeneic	**No •**
D.6.1.3	Xenogeneic	**No •**
D.6.1.3.1	If 'Yes', specify the species of origin:	
D.6.2	Type of cells	
D.6.2.1	Stem cells	**No •**
D.6.2.2	Differentiated cells	**No •**
D.6.2.2.1	If 'Yes', specify the type of cells(e.g. keratinocytes, fibroblasts, chondrocytes, ...):	
D.6.2.3	Others:	**No •**
D.6.2.3.1	If others, specify:	

**D.7**	**PRODUCTS CONTAINING DEVICES (i.e. MEDICAL DEVICES, SCAFFOLDS ETC.)**	
D.7.1	Give a brief description of the device:	
D.7.2	What is the name of the device?	
D.7.3	Is the device implantable?	**No •**
D.7.4	Does this product contain:	
D.7.4.1	A medical device?	**No •**
D.7.4.1.1	Does this medical device have a CE mark?	**No •**
D.7.4.1.1.1	The notified body is:	
D.7.4.2	Bio-materials?	**No •**
D.7.4.3	Scaffolds?	**No •**
D.7.4.4	Matrices?	**No •**
D.7.4.5	Other?	**No •**
D.7.4.5.1	If other, specify:	

D.8 INFORMATION ON PLACEBO (if relevant; repeat as necessary)

D.8.1	Is there a placebo:	**Yes •**
D.8.2	This refers to placebo number:	**PL1**

D.8.3	Pharmaceutical form:	**Capsule**
D.8.4	Route of administration:	**Oral use**
D.8.5	Which IMP is it a placebo for? Specify IMP Number(s) from D.1.1	**PR1**
D.8.5.1	Composition, apart from the active substance(s):	
D.8.5.2	Is it otherwise identical to the IMP?	**No •**
D.8.5.2.1	If not, specify major ingredients: **Lactose**	

D.9 SITE(S) WHERE THE QUALIFIED PERSON CERTIFIES BATCH RELEASE[22]

This section is dedicated to ***finished*** *IMPs, i.e. medicinal products randomised, packaged, labelled and certified for use in the clinical trial. If there is more than one site or more than one IMP is certified, use extra pages and give each IMP its number from section D.1.1 or D.8.2 In the case of multiple sites indicate the product certified by each site*

D.9.1	Do not fill in section D.9.2 for an IMP that:<br>*Has a MA in the EU* ***and***<br>*Is sourced from the EU market* ***and***<br>*Is used in the trial without modification(e.g. not overencapsulated)* ***and***<br>*The packaging and labelling is carried out for local use only as per article 9.2. of the Directive 2005/28/EC (GCP Directive)*<br>If all these conditions are met tick • and list the number(s) of each IMP including placebo from sections D.1.1 and D.8.2 to which this applies<br>**PR1**<br>**PL1**

**D.9.2**	**Who is responsible in the Community for the certification of the finished IMPs?**<br>This site is responsible for certification of (list the number(s) of each IMP including placebo from sections D.1.1 and D.8.2):<br>please tick the appropriate box:	
D.9.2.1	Manufacturer	?
D.9.2.2	Importer	?
D.9.2.3	Name of the organisation:	
D.9.2.4	Address:	
D.9.2.4.1	Street Address	
D.9.2.4.2	Town/City	
D.9.2.4.3	Post Code	
D.9.2.4.4	Country	
D.9.2.5	Give the manufacturing authorisation number:	
D.9.2.5.1	If No authorisation, give the reasons:	

Where the product does not have a MA in the EU, but is supplied in bulk and final packaging and labelling for local use is carried out in accordance with article 9.2 of Directive 2005/28/EC (GCP Directive) then enter the site where the product was finally certified for release by the Qualified Person for use in the clinical trial at D.9.2 above.

E. GENERAL INFORMATION ON THE TRIAL

This section should be used to provide information about the aims, scope and design of the trial. When the protocol includes a sub-study in the MS concerned section E.2.3 should be completed providing information about the sub-study. To identify it check the sub-study box in the 'Objective of the trial' question below.

**E.1**	**MEDICAL CONDITION OR DISEASE UNDER INVESTIGATION**				
E.1.1	Specify the medical condition(s) to be investigated[23] (free text):				
	**English**	**The aim is to measure plasma aldosterone and cortisol concentrations in basal conditions and in response to different stimulation tests of the adrenal function**			
E.1.1.1	Medical condition in easily understood language				
	**English**	**aldosterone and cortisol concentrations in healthy volunteers**			
E.1.1.2	Therapeutic area<br>**Body processes [G] - Physiological processes [G07]**				
E.1.2	MedDRA version, system organ class, level, term and classification code[24]:				
	Version	System Organ Class	Classification Code	Term	Level
E.1.3	Is any of the conditions being studied a rare disease[25]?	**No •**			

**E.2**	**OBJECTIVE OF THE TRIAL**	
E.2.1	Main objective:	
	**English**	**The aim of the study is to use the NK1 receptor antagonist aprepitant as a pharmacological tool to determine the physiological role of the regulation of adrenocortical secretions by the sympathetic system via SP. The influence of the compound on plasma aldosterone and cortisol concentrations will be evaluated in basal conditions and after stimulation.**<br>**This pilot "proof-of-concept" study will allow us verifying that corticosteroid secretions are actually controlled in vivo by a neural stimulatory tone via SP**
E.2.2	Secondary objectives:	
	**English**	**To determine the physiological conditions that involve the regulation of corticosteroid production by tachykinins.**
E.2.3	Is there a sub-study? **No •**	
E.2.3.1	If 'Yes', give the full title, date and version of each sub-study and their related objectives:	

**E.3**	**PRINCIPAL INCLUSION CRITERIA** ***(list the most important)***	
	**English**	**o Male subjects;**<br>**o Age ranging 18 - 30 years old;**<br>**o Submitted to a social security regimen;**<br>**o Agreeing to the study & Informed consent form signed;**<br>**o Body mass index (weight (kg)/height (m)²) < 27;**<br>**o No treatment received 6 weeks before inclusion;**<br>**o No anomaly after: complete clinical examination, pulse and blood pressure measurement, ECG;**<br>**o No biological abnormality after the following biological testing:**<br>**- Hematology: white & red blood cells & platelets count, haemoglobin, hematocrit**<br>**- Blood biochemistry: sodium, potassium, chloride, bicarbonate, creatinine, urea**<br>**- Urinary biochemistry (24 h collection): cortisol, aldosterone**

- Serologies: HIV, HBV, HCV

o No participation in a clinical trial 3 months before inclusion.

**E.4**	**PRINCIPAL EXCLUSION CRITERIA** ***(list the most important)***	
	**English**	**• Subject not agreeing to the study or impossible to follow-up; the persons in detention by judicial or administrative decision, patients hospitalized without consent, individuals admitted to a health or social facility for purposes other than research and legally protected adults or not in a position to express their consent.**<br>**• Known history of significant medical or surgical pathology, notably endocrine;**<br>**• Renal or hepatic insufficiency;**<br>**• Nephrotic syndrome;**<br>**• Edematous syndrome;**<br>**• Hypertension or postural hypotension;**<br>**• Cardiac rhythm or conduction pathologies;**<br>**• Cardiac insufficiency;**<br>**• Epilepsy;**<br>**• Significant psychiatric disorder;**<br>**• Known history of severe allergy, hypersensitivity to aprepitant and/or metoclopramide;**<br>**• Hereditary problems of fructose intolerance, glucose-galactose malabsorption or sucrase-isomaltase deficit;**<br>**• Impaired lactose tolerance.**

**E.5**	**END POINT(S):**	
E.5.1	Primary End Point (repeat as necessary)[26]	
	**English**	**Plasma aldosterone levels during orthostatic test**
E.5.1.1	Timepoint(s) of evaluation of this end point	
	**English**	**Day 4 of treatment**
E.5.2	Secondary End Point (repeat as necessary)	
	**English**	**Basal aldosterone alteration, aldosterone variation during metoclopramide and hypoglycaemia tests.**<br>**Basal and stimulated alterations of renin, cortisol and ACTH during 3 different stimulatory tests (upright, metoclopramide and hypoglycaemia tests)**
E.5.2.1	Timepoint(s) of evaluation of this end point	
	**English**	**Day 4, day 5 and day 7 of treatment**

**E.6**	**SCOPE OF THE TRIAL – Tick all boxes where applicable**	
E.6.1	Diagnosis	**No •**
E.6.2	Prophylaxis	**No •**
E.6.3	Therapy	**No •**
E.6.4	Safety	**No •**
E.6.5	Efficacy	**No •**
E.6.6	Pharmacokinetic	**No •**
E.6.7	Pharmacodynamic	**No •**
E.6.8	Bioequivalence	**No •**
E.6.9	Dose Response	**No •**
E.6.10	Pharmacogenetic	**No •**

E.6.11	Pharmacogenomic	**No •**
E.6.12	Pharmacoeconomic	**No •**
E.6.13	Others	**Yes •**
E.6.13.1	If others, specify:	
	**English** **Physiological study**	

**E.7**	**TRIAL TYPE AND PHASE[27]**	
E.7.1	Human pharmacology (Phase I)	**No •**
Is it:		
E.7.1.1	First administration to humans	**No •**
E.7.1.2	Bioequivalence study	**No •**
E.7.1.3	Other:	**No •**
E.7.1.3.1	If other, please specify:	
E.7.2	Therapeutic exploratory (Phase II)	**No •**
E.7.3	Therapeutic confirmatory (Phase III)	**No •**
E.7.4	Therapeutic use(Phase IV)	**Yes •**

**E.8**	**DESIGN OF THE TRIAL**	
E.8.1	Controlled	**Yes •**
	If 'Yes', specify:	
E.8.1.1	Randomised:	**Yes •**
E.8.1.2	Open:	**No •**
E.8.1.3	Single blind:	**No •**
E.8.1.4	Double blind:	**Yes •**
E.8.1.5	Parallel group:	**No •**
E.8.1.6	Cross over:	**Yes •**
E.8.1.7	Other:	**No •**
E.8.1.7.1	If other specify:	
E.8.2	If controlled, specify the comparator:	
E.8.2.1	Other medicinal product(s)	**No •**
E.8.2.2	Placebo	**Yes •**
E.8.2.3	Other	**No •**
E.8.2.3.1	If 'Yes' to other, specify :	
E.8.2.4	Number of treatment arms in the trial	**2**
E.8.3	Single site in the Member State concerned (see also section G):	**Yes •**
E.8.4	Multiple sites in the Member State concerned(see also section G):	**No •**
E.8.4.1	Number of sites anticipated in Member State concerned	
E.8.5	Multiple Member States:	**No •**
E.8.5.1	Number of sites anticipated in the EEA:	
E.8.6	Trial involving sites outside the EEA:	
E.8.6.1	Trial being conducted both within and outside the EEA:	**No •**
E.8.6.2	Trial being conducted completely outside of the EEA:	**No •**
E.8.6.3	If E.8.6.1 or E.8.6.2 are Yes, specify the regions in which trial sites are planned:	
E.8.6.4	If E.8.6.1 or E.8.6.2 are Yes, specify the number of sites anticipated outside of the EEA:	
E.8.7	Trial having an independent data monitoring committee:	**No •**
E.8.8	Definition of the end of trial: If it is the last visit of the last subject, please enter "LVLS". If it is not LVLS provide the definition:	
	**English** **LVLS**	
E.8.9	Initial estimate of the duration of the trial[28] (years, months and days)	
E.8.9.1	In the Member State concerned	**years 13 months days**
E.8.9.2	In all countries concerned by the trial	**years 13 months days**
E.8.10	Proposed date of start of recruitment	
E.8.10.1	In the Member State concerned	
E.8.10.2	In any country	

F. POPULATION OF TRIAL SUBJECTS

**F.1**	**AGE RANGE**		
F.1.1	Are the trial subjects under 18?		**No •**
	If 'Yes', specify the estimated number of subjects planned in each age range for the whole trial:	Approx. No. of patients[29]	
F.1.1.1	In utero	()	**No •**
F.1.1.2	Preterm newborn infants (up to gestational age < 37 weeks)	()	**No •**
F.1.1.3	Newborns (0-27 days)	()	**No •**
F.1.1.4	Infants and toddlers (28 days - 23 months)	()	**No •**
F.1.1.5	Children (2-11 years)	()	**No •**
F.1.1.6	Adolescents (12-17 years)	()	**No •**
F.1.2	Adults (18-64 years)	(**20**)	**Yes •**
F.1.3	Elderly (>= 65 years)	()	**No •**

**F.2**	**GENDER**	
F.2.1	Female	**No •**
F.2.2	Male	**Yes •**

**F.3**	**GROUP OF TRIAL SUBJECTS**	
F.3.1	Healthy volunteers	**Yes •**
F.3.2	Patients	**No •**
F.3.3	Specific vulnerable populations	**No •**
F.3.3.1	Women of child bearing potential not using contraception	**No •**
F.3.3.2	Women of child bearing potential using contraception	**No •**
F.3.3.3	Pregnant women	**No •**
F.3.3.4	Nursing women	**No •**
F.3.3.5	Emergency situation	**No •**
F.3.3.6	Subjects incapable of giving consent personally	**No •**
F.3.3.6.1	If 'Yes', specify:	
F.3.3.7	Others:	**No •**
F.3.3.7.1	If 'Yes', specify:	

**F.4**	**PLANNED NUMBER OF SUBJECTS TO BE INCLUDED:**	
F.4.1	In the member state	**20**
F.4.2	For a multinational trial**:**	
F.4.2.1	In the EEA	
F.4.2.2	In the whole clinical trial	

**F.5**	**PLANS FOR TREATMENT OR CARE AFTER THE SUBJECT HAS ENDED HIS/HER PARTICIPATION IN THE TRIAL. please specify** (free text):
	**English** **None**

G. CLINICAL TRIAL SITES/INVESTIGATORS IN THE MEMBER STATE CONCERNED BY THIS REQUEST

**G.1**	**CO-ORDINATING INVESTIGATOR (for multicentre trial) and principal investigator (for single centre trial)**	
G.1.1	Given name:	**Hervé**
G.1.2	Middle name, if applicable:	
G.1.3	Family name:	**LEFEBVRE**
G.1.4	Qualification (MD.........)	**MD, PhD**
G.1.5	Professional address:	
G.1.5	Institution name	**CHU de Rouen**
G.1.5	Institution department	**Service d'Endocrinologie, Diabète et Maladies Métaboliques**
G.1.5.1	Street address	
G.1.5.2	Town/city	**Rouen**
G.1.5.3	Post code	
G.1.5.4	Country	**France**
G.1.6	Telephone number:	
G.1.7	Fax number:	
G.1.8	E-mail:	

**G.2**	**PRINCIPAL INVESTIGATORS *(for multicentre trial ; where necessary, use additional forms)***
G.2.1	Given name:
G.2.2	Middle name, if applicable:
G.2.3	Family name:
G.2.4	Qualification (MD.........)
G.2.5	Professional address:
G.2.5	Institution name
G.2.5	Institution department
G.2.5.1	Street address
G.2.5.2	Town/city
G.2.5.3	Post code
G.2.5.4	Country
G.2.6	Telephone number:
G.2.7	Fax number:
G.2.8	E-mail:

**G.3**	**CENTRAL TECHNICAL FACILITIES TO BE USED IN THE CONDUCT OF THE TRIAL**<br><br>**Laboratory or other technical facility, in which the measurement or assessment of the main evaluation criteria are centralised** (repeat as needed for multiple organisations).	
G.3.1	Name of organisation:	**Institut de Biologie Clinique (IBC)**
G.3.2	Department	**CHU de Rouen**
G.3.3	Name of contact person:	
G.3.3.1	Given name	**Jean-François**
G.3.3.2	Middle name	
G.3.3.3	Family name	**LEMELAND**
G.3.4	Address:	
G.3.4.1	Street address	
G.3.4.2	Town/city	
G.3.4.3	Post code	
G.3.4.4	Country	
G.3.5	Telephone number:	
G.3.6	Fax number:	
G.3.7	E-mail:	
G.3.8	Enter the details of any duties subcontracted to this central technical facility in this trial	

G.3.8.1	Routine clinical pathology testing	**No •**
G.3.8.2	Clinical chemistry	**Yes •**
G.3.8.3	Clinical haematology	**Yes •**
G.3.8.4	Clinical microbiology	**No •**
G.3.8.5	Histopathology	**No •**
G.3.8.6	Serology/ endocrinology	**Yes •**
G.3.8.7	Analytical chemistry	**No •**
G.3.8.8	ECG analysis/ review	**No •**
G.3.8.9	Medical image analysis/ review - X-ray, MRI, ultrasound, etc.	**No •**
G.3.8.10	Primary/ surrogate endpoint test	**No •**
G.3.8.11	Other Duties subcontracted?	**No •**
G.3.8.11.1	If 'Yes', specify the other duties	

G.3 CENTRAL TECHNICAL FACILITIES TO BE USED IN THE CONDUCT OF THE TRIAL

Laboratory or other technical facility, in which the measurement or assessment of the main evaluation criteria are centralised (repeat as needed for multiple organisations).

G.3.1	Name of organisation:	**Centre d'Investigation Clinique**
G.3.2	Department	**CHU de Rouen**
G.3.3	Name of contact person:	
G.3.3.1	Given name	**Jacques**
G.3.3.2	Middle name	
G.3.3.3	Family name	**WEBER**
G.3.4	Address:	
G.3.4.1	Street address	
G.3.4.2	Town/city	
G.3.4.3	Post code	
G.3.4.4	Country	
G.3.5	Telephone number:	
G.3.6	Fax number:	
G.3.7	E-mail:	
G.3.8	Enter the details of any duties subcontracted to this central technical facility in this trial	
G.3.8.1	Routine clinical pathology testing	**No •**
G.3.8.2	Clinical chemistry	**No •**
G.3.8.3	Clinical haematology	**No •**
G.3.8.4	Clinical microbiology	**No •**
G.3.8.5	Histopathology	**No •**
G.3.8.6	Serology/ endocrinology	**No •**
G.3.8.7	Analytical chemistry	**No •**
G.3.8.8	ECG analysis/ review	**Yes •**
G.3.8.9	Medical image analysis/ review - X-ray, MRI, ultrasound, etc.	**No •**
G.3.8.10	Primary/ surrogate endpoint test	**No •**
G.3.8.11	Other Duties subcontracted?	**Yes •**
G.3.8.11.1	If 'Yes', specify the other duties	**clinical study central site**

G.4 NETWORKS TO BE INVOLVED IN THE TRIAL (e.g. Paediatric Networks involved in the trial)

G.4.1	Name of organisation:
G.4.2	Name of contact person:
G.4.2.1	Given name
G.4.2.2	Middle name
G.4.2.3	Family name
G.4.3	Address:
G.4.3.1	Street address
G.4.3.2	Town/city
G.4.3.3	Post code
G.4.3.4	Country

G.4.4	Telephone number:	
G.4.5	Fax number:	
G.4.6	E-mail:	
G.4.7	Activities carried out by the network:	

**G.5**	**ORGANISATIONS TO WHOM THE SPONSOR HAS TRANSFERRED TRIAL RELATED DUTIES AND FUNCTIONS**	
G.5.1	**Has the sponsor transferred any major or all the sponsor's trial related duties and functions to another organisation or third party?**	**No •**
Repeat as necessary for multiple organisations:		
G.5.1.1	Organisation name:	
G.5.1.2	Organisation department	
G.5.1.3	Name of contact person :	
G.5.1.3.1	Given name	
G.5.1.3.2	Middle name	
G.5.1.3.3	Family name	
G.5.1.4	Address:	
G.5.1.4.1	Street address	
G.5.1.4.2	Town/city	
G.5.1.4.3	Post code	
G.5.1.4.4	Country	
G.5.1.5	Telephone number:	
G.5.1.6	Fax number:	
G.5.1.7	E-mail:	
G.5.1.8	All tasks of the sponsor	**Yes ? No ? Not Answered ?**
G.5.1.9	Monitoring	**Yes ? No ? Not Answered ?**
G.5.1.10	Regulatory (e.g. preparation of applications to CA and ethics committee)	**Yes ? No ? Not Answered ?**
G.5.1.11	Investigator recruitment	**Yes ? No ? Not Answered ?**
G.5.1.12	IVRS[30] – treatment randomisation	**Yes ? No ? Not Answered ?**
G.5.1.13	Data management	**Yes ? No ? Not Answered ?**
G.5.1.14	E-data capture	**Yes ? No ? Not Answered ?**
G.5.1.15	SUSAR reporting	**Yes ? No ? Not Answered ?**
G.5.1.16	Quality assurance auditing	**Yes ? No ? Not Answered ?**
G.5.1.17	Statistical analysis	**Yes ? No ? Not Answered ?**
G.5.1.18	Medical writing	**Yes ? No ? Not Answered ?**
G.5.1.19	Other duties subcontracted?	**Yes ? No ? Not Answered ?**
G.5.1.19.1	If 'Yes' to other, please specify:	

H. COMPETENT AUTHORITY / ETHICS COMMITTEE IN THE MEMBER STATE CONCERNED BY THIS REQUEST

**H.1**	**TYPE OF APPLICATION**	
If this application is addressed to the Competent Authority, please tick the Ethics Committee box and give information on the Ethics committee concerned. If this application is addressed to the Ethics Committee, please tick the Competent Authority box and give the information on the Competent Authority concerned.		
H.1.1	Competent Authority	**No •**
H.1.2	Ethics Committee	**Yes •**

**H.2**	**INFORMATION ON ETHICS COMMITTEE**	
H.2.1	Name:	**CPP Nord-Ouest I**
H.2.2	Address	
H.2.2.1	Street address	
H.2.2.2	Town/city	**Rouen**
H.2.2.3	Post code	**76031**
H.2.2.4	Country	**France**
H.2.3	Date of submission:	**2008-10-21**

**H.3**	**OPINION**	
H.3.1	To be requested	**No •**
H.3.2	Pending	**No •**
H.3.3	Given	**Yes •**
	If 'Given', specify:	
H.3.3.1	Date of opinion:	**2008-12-18**
H.3.3.2	Opinion favourable	**Yes •**
H.3.3.3	Opinion not favourable	**No •**
	If not favourable, give:	
H.3.3.3.1	The reasons	
H.3.3.3.2	The eventual anticipated date of resubmission:	

I. SIGNATURE OF THE APPLICANT IN THE MEMBER STATE

**I.1**	I hereby confirm that /confirm on behalf of the sponsor (delete which is not applicable) that:<br>• the information provided is complete;<br>• the attached documents contain an accurate account of the information available;<br>• the clinical trial will be conducted in accordance with the protocol; and<br>• the clinical trial will be conducted, and SUSARs and result-related information will be reported, in accordance with the applicable legislation.

**I.2**	**APPLICANT OF THE REQUEST FOR THE COMPETENT AUTHORITY** (as stated in section C.1):
I.2.1	Date:
I.2.2	Signature[31]:
I.2.3	Print name:

**I.3**	**APPLICANT OF THE REQUEST FOR THE ETHICS COMMITTEE** (as stated in section C.2):
I.3.1	Date:
I.3.2	Signature[32]:
I.3.3	Print name:

ENDNOTES

[1] Any translation of the protocol should be assigned the same date and version as those in the original document.
[2] International Standard Randomised Controlled Trial Number. Sponsors may wish to use an International Standardised Random Controlled Trial Number (ISRCTN) to identify their trial in addition to the EudraCT number; for instance if their trial is part of a multinational trial with sites outside the Community. They can obtain the number and guidance from the Current Controlled Trials website http://www.controlled-trials.com/isrctn to which there is a link from the EudraCT database website http://eudract.ema.europa.eu. When available they should provide it in Section A.6 of the application form.
[3] US National Clinical Trial (NCT) Numbers required on the FDA clinical trial application form.
[4] For a resubmission following previous withdrawal of an application or unfavourable opinion of an ethics committee, or previous withdrawal of an application or refusal of a request by the competent authority, enter a letter in the sequence, A for first resubmission, B for second, C for third et seq.
[5] In accordance with Article 19 of Directive 2001/20/EC.
[6] The contact point should give functional information rather than details of one "person", in order to avoid the need for update and maintenance of these contact details.
[7] This requires a EudraLink account. (See https://eudract.ema.europa.eu/document.html for details)
[8] According to national legislation.
[9] Available from the Summary of Product Characteristics (SmPC)
[10] According to the Community register on orphan medicinal products (Regulation (EC) n° 141/2000): http://ec.europa.eu/enterprise/pharmaceuticals/register/index.htm
[11] Committee for Medicinal Products for Human Use of the European Medicines Agency
[12] To be provided only when there is No trade name. This is the name routinely used by a sponsor to identify the IMP in the CT documentation (protocol, IB…).
[13] To be provided only when there is No trade name. This is a code designated by the sponsor which represents the name routinely used by the sponsor to identify the product in the CT documentation. For example, a code may be used for combinations of drugs or drugs and devices.
[14] Available from the Summary of Product Characteristics (SmPC).
[15] Chemical Abstracts Service.
[16] Complete also section D.4 Cell therapy as defined in Annex 1 part IV of Directive 2001/83/EC as amended.
[17] Complete also section D.5 Gene Therapy as defined in Annex 1 part IV of Directive 2001/83/EC as amended.
[18] Complete also section D.6 - Tissue Engineered Product as defined in Article 2(1)(b) of Regulation1394/2007/EC.
[19] Complete also section D.7
[20] The mode of action should briefly describe the chemical, biochemical, immunological or biological means the IMP uses to effect its pharmaceutical action.
[21] Guideline on strategies to identify and mitigate risks for first-in-human clinical trials with investigational medicinal products. EMEA/CHMP/SWP/28367/2007 19 July 2007
[22] In accordance with paragraph 38 of Annex 13 of Volume 4 of the Rules Governing Medical Products in the European Union.
[23] In the case of healthy volunteer trials, the intended indication for the product under development should be provided.
[24] Applicants are encouraged to provide the MedDRA lower level term if applicable and classification code. These can be accessed from the EMEA EudraCT website (http://eudract.ema.europa.eu/).
[25] Points to consider on the calculation and reporting of the prevalence of a condition for Orphan drug designation: COM/436/01 (http://www.ema.europa.eu/htms/human/orphans/intro.htm).
[26] The protocol will usually identify a single primary end point but there may be a co-primary end point in some cases and/or a number of secondary end points.
[27] The descriptions of the trial types provided are those recommended in preference to Phases. See page 5 of Community guideline CPMP/ICH/291/95. The development of a new indication after initial approval of a medicine should be considered as a new development plan.
[28] From the first inclusion until the last visit of the last subject.
[29] These numbers will be initial estimates. Applicants will not be required to update this information nor do they constitute an authorisation or restriction on the inclusion of these numbers of patients in the trial. The numbers of subjects whose inclusion is authorised are those set out in the authorised version of the protocol, or subsequent authorised amendments.
[30] Interactive Voice Response System: commonly used for randomisation of treatment and controlling the shipment of stock of product.
[31] On an application to the Competent Authority only, the applicant to the Competent Authority needs to sign.

[32] On an application to the Ethics Committee only, the applicant to the Ethics Committee needs to sign.

Supplementary References

1. H. Lefebvre *et al.*, Serotonin-induced stimulation of cortisol secretion from human adrenocortical tissue is mediated through activation of a serotonin4 receptor subtype. *Neuroscience*. **47**, 999–1007 (1992).
2. H. Lefebvre *et al.*, Production and metabolism of serotonin (5-HT) by the human adrenal cortex: paracrine stimulation of aldosterone secretion by 5-HT. *J. Clin. Endocrinol. Metab.* **86**, 5001–5007 (2001).
3. C. Duparc *et al.*, Mast cell hyperplasia is associated with aldosterone hypersecretion in a subset of aldosterone-producing adenomas. *J. Clin. Endocrinol. Metab.* **100**, E550-560 (2015).
4. H. Lefebvre *et al.*, Effect of the serotonin-4 receptor agonist zacopride on aldosterone secretion from the human adrenal cortex: in vivo and in vitro studies. *J. Clin. Endocrinol. Metab.* **77**, 1662–1666 (1993).
5. H. Lefebvre *et al.*, The serotonin-4 receptor agonist cisapride and angiotensin-II exert additive effects on aldosterone secretion in normal man. *J. Clin. Endocrinol. Metab.* **80**, 504–507 (1995).
6. J.H. Pratt, A. Ganguly, C.A. Parkinson, M.H.Weinberger, Stimulation of aldosterone secretion by metoclopramide in humans: apparent independence of renal and pituitary mediation. *Metabolism* **30**,129–134 (1981).
7. E. P. Bouras, M. Camilleri, D. D. Burton, S. McKinzie, Selective stimulation of colonic transit by the benzofuran 5HT4 agonist, prucalopride, in healthy humans. *Gut*. **44**, 682–686 (1999).
8. Gomez-Sanchez, C. E. *et al.* Development of monoclonal antibodies against human CYP11B1 and CYP11B2. *Mol. Cell. Endocrinol.* **383**, 111–117 (2014).

9. Christensen, J., Alfredson, H. & Andersson, G. Protease-activated receptors in the Achilles tendon-a potential explanation for the excessive pain signalling in tendinopathy. *Mol. Pain* **11**, 13 (2015).

10. García-Ortega, J. *et al.* Expression of Tachykinins and Tachykinin Receptors and Interaction with Kisspeptin in Human Granulosa and Cumulus Cells. *Biol. Reprod.* **94**, 124 (2016).

11. Ortiz-Prieto, A., Bernabeu-Wittel, J., Zulueta-Dorado, T., Lorente-Lavirgen, A. I. & Muñoz, M. Immunolocalization of substance P and NK-1 receptor in vascular anomalies. *Arch. Dermatol. Res.* **309**, 97–102 (2017).

12. Peirs, C. *et al.* Dorsal Horn Circuits for Persistent Mechanical Pain. *Neuron* **87**, 797–812 (2015).

13. Yackle, K. *et al.* Breathing control center neurons that promote arousal in mice. *Science* **355**, 1411–1415 (2017).

14. Leonard, A. V., Manavis, J., Blumbergs, P. C. & Vink, R. Changes in substance P and NK1 receptor immunohistochemistry following human spinal cord injury. *Spinal Cord* **52**, 17–23 (2014).

15. Yuan, S.-B. *et al.* Gp120 in the pathogenesis of human immunodeficiency virus-associated pain. *Ann. Neurol.* **75**, 837–850 (2014).

16. Rohn, T. T. & Catlin, L. W. Immunolocalization of influenza A virus and markers of inflammation in the human Parkinson's disease brain. *PloS One* **6**, e20495 (2011).

17. Hoover, D. B. *et al.* Localization of multiple neurotransmitters in surgically derived specimens of human atrial ganglia. *Neuroscience* **164**, 1170–1179 (2009).
