# Supplementary material for: The neuropeptide substance P regulates aldosterone secretion in human adrenals
Source: Nat Commun. 2020 May 29;11:2673. doi: 10.1038/s41467-020-16470-8 (PMC7260184; doi:10.1038/s41467-020-16470-8)
Supplement: Supplementary file 3 — Reporting Summary [file 41467_2020_16470_MOESM3_ESM.pdf]

nature research

Corresponding author(s): H. Lefebvre

Last updated by author(s): Mar 18, 2020

Reporting Summary

Nature Research wishes to improve the reproducibility of the work that we publish. This form provides structure for consistency and transparency in reporting. For further information on Nature Research policies, see Authors & Referees and the Editorial Policy Checklist .

Statistics

For all statistical analyses, confirm that the following items are present in the figure legend, table legend, main text, or Methods section.

n/a	Confirmed	
☐	☒	The exact sample size (*n*) for each experimental group/condition, given as a discrete number and unit of measurement
☐	☒	A statement on whether measurements were taken from distinct samples or whether the same sample was measured repeatedly
☐	☒	The statistical test(s) used AND whether they are one- or two-sided<br>*Only common tests should be described solely by name; describe more complex techniques in the Methods section.*
☒	☐	A description of all covariates tested
☒	☐	A description of any assumptions or corrections, such as tests of normality and adjustment for multiple comparisons
☐	☒	A full description of the statistical parameters including central tendency (e.g. means) or other basic estimates (e.g. regression coefficient) AND variation (e.g. standard deviation) or associated estimates of uncertainty (e.g. confidence intervals)
☐	☒	For null hypothesis testing, the test statistic (e.g. *F*, *t*, *r*) with confidence intervals, effect sizes, degrees of freedom and *P* value noted<br>*Give P values as exact values whenever suitable.*
☒	☐	For Bayesian analysis, information on the choice of priors and Markov chain Monte Carlo settings
☒	☐	For hierarchical and complex designs, identification of the appropriate level for tests and full reporting of outcomes
☒	☐	Estimates of effect sizes (e.g. Cohen's *d*, Pearson's *r*), indicating how they were calculated

Our web collection on statistics for biologists contains articles on many of the points above.

Software and code

Policy information about availability of computer code

Data collection	Real time RT-PCR: QuantStudio 3 system, ThermoFischer<br>Immunohistology: TCS SP8 MP confocal microscope (Leica) with Las X software<br>Microfluorometry: Flexstation III (Molecular Devices, Sunnyvale, CA) with the SoftMax Pro v.5.4 software<br>Western Blot: ChemiDoc Imaging Systems (Bio-Rad) with Image lab software (Bio-Rad).<br>Hormone assays: Immulite 2500 (DPC, La Garenne-Colombes, France), Gamma Counter Wallac Wizard 1470 (Perkin Elmer, Villebon-sur-Yvette, France), Synchron LXi 725 (Beckman Coulter, Villepinte, France)
Data analysis	Microsoft Office Excel software 97-2003<br>Prism 6, GraphPad Software Inc.<br>NCSS, Hintze J, Kaysville, UT<br>StatXact-8 Crossover (Cytel Inc. Software, Antony, France)

For manuscripts utilizing custom algorithms or software that are central to the research but not yet described in published literature, software must be made available to editors/reviewers. We strongly encourage code deposition in a community repository (e.g. GitHub). See the Nature Research guidelines for submitting code & software for further information.

Data

Policy information about availability of data

All manuscripts must include a data availability statement. This statement should provide the following information, where applicable:

- Accession codes, unique identifiers, or web links for publicly available datasets
- A list of figures that have associated raw data
- A description of any restrictions on data availability

The quantitative data that support the findings of the in vitro studies illustrated in figures 1A, 2A, 3A-D, 4A-B, 5B-F, and Supplementary Fig. 5 are available via the following link code https://zenodo.org/record/3736387#.XoSXUIgzaUk (DOI 10.5281/zenodo.3736386). The data that support the findings of the clinical study are

available from the corresponding author (HL) on reasonable request. These data are not publicly available due to them containing information that could compromise research participant privacy/consent.

Field-specific reporting

Please select the one below that is the best fit for your research. If you are not sure, read the appropriate sections before making your selection.

- [x] Life sciences
- [ ] Behavioural & social sciences
- [ ] Ecological, evolutionary & environmental sciences

For a reference copy of the document with all sections, see nature.com/documents/nr-reporting-summary-flat.pdf

Life sciences study design

All studies must disclose on these points even when the disclosure is negative.

Sample size

For in vitro studies, considering the fact that there was no former available data with substance P that could have helped us to estimate the sizes of our sample sets, the sample sizes were defined on the basis of previous experiments performed in our lab for the study of serotonin which acts as a paracrine adrenal factor to stimulate corticosteroid production [Duparc et al. Horm Metab Res. 49, 269-275 (2017); Bram et al JCI Insight 1(15):e87958 (2016); Louiset et al. Mol Cell Endocrinol. 5;441:99-107 (2017)], .
The clinical study is also totally original and has thus no previous equivalent in the literature. It was thus impossible to accurately calculate the size of the required population. We have therefore used a population size similar to that used in previous short proof-of-concept trials which successfully demonstrated an effect of pharmacological agents targeted to serotonin receptors on corticosteroid production in healthy volunteers [Bharucha, A. E. et al. Gut 47, 667–674 (2000); Duparc et al. Horm Metab Res. 49, 269-275 (2017)]. We have also chosen to use a cross-over design to increase the statistical power of our trial.

Data exclusions

For in vitro functional studies, exclusion criteria of data were pre-established as follows:
- absence of any response to both test substances (tachykinins) and positive controls (angiotensin II, ATP or KCl).
- negative responses to prolonged treatment (24h) of adrenal cells with substance P since such data may possibly result from peptide degradation.

For the in vivo study, the exclusion criteria were the following, as listed in the clinical trial protocol:
- Subject not agreeing to the study or impossible to follow-up
- The persons in detention by judicial or administrative decision, patients hospitalized without consent, individuals admitted to a health or social facility for purposes other than research and legally protected adults or not in a position to express their consent.
- Known history of significant medical or surgical pathology, notably endocrine
- Renal or hepatic insufficiency
- Nephrotic syndrome
- Edematous syndrome
- Hypertension or postural hypotension
- Cardiac rhythm or conduction pathologies
- Cardiac insufficiency
- Epilepsy
- Significant psychiatric disorder
- Known history of severe allergy, hypersensitivity to aprepitant and/or metoclopramide
- Hereditary problems of fructose intolerance, glucose-galactose malabsorption or sucrase-isomaltase deficit
- Impaired lactose tolerance.

In addition, a single measurement of 24-h urine aldosterone excretion failed in one volunteer and was thus excluded for statistical analysis.

Replication

For in vitro studies, all data were replicated independently at least three times, by different investigators and using different batches of reactives and test substances.
For the clinical study, each blood sample was assayed in duplicate. The two samples were re-assayed when the variation coefficient between the two values of each pair of data was higher than 10%.

Randomization

For in vitro functional studies, samples were randomly allocated to treatment groups.
For the in vivo study, all healthy volunteers enrolled in the study underwent the two treatment periods, i.e. placebo and aprepitant. The order of the two periods was randomly assigned for each volunteer.

Blinding

For in vitro studies, blinding was used for hormone assays. Blinding was unrelevant for RT-qPCR and immunohistological studies which were applied on normal adrenal samples without any test treatment.
The clinical trial was a double-blind study.

Reporting for specific materials, systems and methods

We require information from authors about some types of materials, experimental systems and methods used in many studies. Here, indicate whether each material, system or method listed is relevant to your study. If you are not sure if a list item applies to your research, read the appropriate section before selecting a response.

Materials & experimental systems

n/a	Involved in the study	
☐	☒	Antibodies
☒	☐	Eukaryotic cell lines
☒	☐	Palaeontology
☒	☐	Animals and other organisms
☐	☒	Human research participants
☐	☒	Clinical data

Methods

n/a	Involved in the study	
☒	☐	ChIP-seq
☒	☐	Flow cytometry
☒	☐	MRI-based neuroimaging

Antibodies

Antibodies used

Aldosterone Synthase: Monoclonal mouse anti-human CYP11B2 clone 41.
Substance P: ref MM-0001-1 (Interchim) clone NC1/34.
NK1 receptor: ref T5950 (Sigma-Aldrich).
NK1 receptor: ref PA3-301 (ThermoFisher).
NK1 receptor: ref SAB4502913 (Sigma-Aldrich).
PGP9.5: ref AB1761 (Merck).
Tyrosine hydroxylase: ref MAB318, clone LNC1 (Merck Millipore).
Choline acetyl transferase: ref AB144P (Merck Millipore).
p44/42 MAPK (ERK1/2): ref M5670 (Sigma-Aldrich).
phospho-p44/42 MAPK (ERK1/2): ref 4370 (Cell signaling).
Vinculine: ref MCA465GA (Biorad).

Validation

Aldosterone Synthase: Monoclonal mouse anti-human CYP11B2 clone 41 validated on human adrenal (Gomez-Sanchez C et al, Mol Cell Endocrinol. 2014 ;383(1-2):111-7).
Substance P: ref MM-0001-1 (Interchim) clone NC1/34 validated on human Achilles tendon (Christensen J et al, Mol Pain. 2015 11:13)
NK1 receptor: ref T5950 (Sigma-Aldrich) valided on human ovary (García-Ortega J et al, Biol Reprod. 2016 ;94:124).
NK1 receptor: ref PA3-301 (ThermoFisher) validated on human skin and pancreatic carcinoma (https://www.thermofisher.com/antibody/product/TACR1-Antibody-Polyclonal/PA3-301)
NK1 receptor: ref SAB4502913 (Sigma-Aldrich) validated on human blood vessel (Ortiz-Prieto A et al. Arch. Dermatol. Res. 2017,309:97–102) and mouse neurons (Peirs C et al. Neuron 2015;87:797–812; Yackle K et al. Science 2017;355:1411–1415) (https://www.sigmaaldrich.com/catalog/product/sigma/sab4502913?lang=fr&region=FR).
PGP9.5: ref AB1761 (Merck) validated on human spinal cord (Yuan SB et al. Ann. Neurol. 2014; 75: 837–850) (http://www.merckmillipore.com/FR/fr/product/Anti-Protein-Gene-Product-9.5-Antibody,MM_NF-AB1761).
Tyrosine hydroxylase: ref MAB318, clone LNC1 (Merck Millipore) validated in substantia nigra from human brain (Rohn TT, Catlin LW., PLoS One. 2011;6(5):e20495)
Choline acetyl transferase: ref AB144P (Merck Millipore) validated on human cardiac neurons (Hoover DB et al, Neuroscience 2009, 164, 1170) and placenta lysates (http://www.merckmillipore.com/FR/fr/product/Anti-Choline-Acetyltransferase-Antibody,MM_NF-AB144P)
p44/42 MAPK (ERK1/2): ref M5670 (Sigma-Aldrich) validated on different cell lines (https://www.sigmaaldrich.com/catalog/product/sigma/m5670?lang=fr&region=FR)
phospho-p44/42 MAPK (ERK1/2): ref 4370 (Cell signaling) validated on different cell lines (https://www.cellsignal.com/products/primary-antibodies/phospho-p44-42-mapk-erk1-2-thr202-tyr204-d13-14-4e-xp-rabbit-mab/4370)
Vinculine: ref MCA465GA (Biorad) validated on Hela cells (https://www.bio-rad-antibodies.com/monoclonal/human-vinculin-antibody-v284-mca465.html?f=purified)

Human research participants

Policy information about studies involving human research participants

Population characteristics	20 Male subjects<br>Age 21.3±1.7 year<br>Body mass index 22.4±1.7 kg/m2<br>Systolic blood pressure 127.9±11.3 mmHg<br>Diastolic blood pressure 69.3±7.4 mmHg<br>Cardiac frequency 70±11 bpm
Recruitment	Healthy subjects were recruited by the Centre for Clinical Investigation of the Rouen University Hospital from the institutional register of volunteers for clinical research. In addition, the absence of intercurrent treatment and potential subclinical adrenal disease has been verified prior to inclusion of the subjects. There is thus no potential bias.
Ethics oversight	The study was approved by the Institutional Review Board of the University Hospital of Rouen, the regional Ethics Committee (Comité de Protection des Personnes de Haute-Normandie) and the French National Agency for Biomedicine (Agence de Biomédecine).

Note that full information on the approval of the study protocol must also be provided in the manuscript.

Clinical data

Policy information about clinical studies

All manuscripts should comply with the ICMJE guidelines for publication of clinical research and a completed CONSORT checklist must be included with all submissions.

Clinical trial registration	Protocol n° 2007/049/HP (APHOS study); EudraCT: 2008-003367-40; ClinicalTrial.gov: NCT00977223
Study protocol	Available at ClinicalTrials.gov and EudraCT at clinicaltrialsregister.eu
Data collection	June 2009 to June 2010 by the Centre for Clinical Investigation of the Rouen University Hospital
Outcomes	Primary outcomes: aldosterone secretion in recumbent and upright positions assessed by plasma aldosterone levels.<br><br>Secondary outcomes: Aldosterone variation during metoclopramide & hypoglycaemia tests (basal and stimulated), variations of plasma renin, cortisol & ACTH levels. Daily aldosterone and cortisol productions assessed by urinary aldosterone and cortisol.
